# Supplementary material for: Social Media Exposure and Muscle Dysmorphia Risk in Young German Athletes: A Cross-Sectional Survey with Machine-Learning Insights Using the MDDI-1
Source: Healthcare (Basel). 2025 Jul 15;13(14):1695. doi: 10.3390/healthcare13141695 (PMC12294503; doi:10.3390/healthcare13141695)
Supplement: Supplementary file 1 [file healthcare-13-01695-s001.zip › healthcare-3684697-supplementary.pdf]

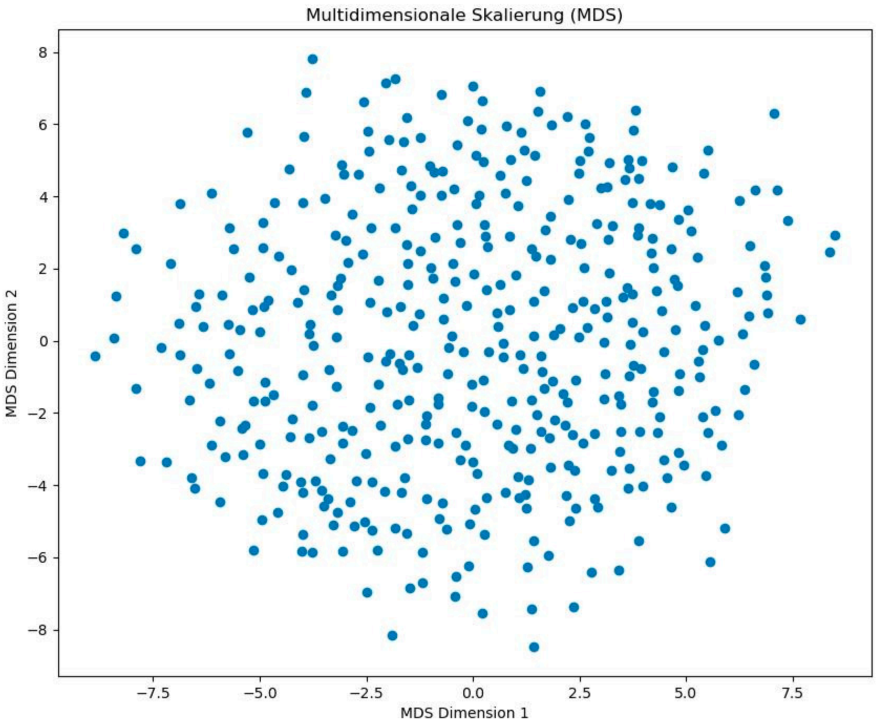

Supplementary Figure S1. Visualization of variables using Multidimensional Scaling: Multidimensional Scaling (MDS) was employed to visualize the high-dimensional relationships in our dataset, reducing it to two dimensions for clarity insights into the intricate connections between

variables related to demographics, muscle training, body satisfaction, and social media activities.

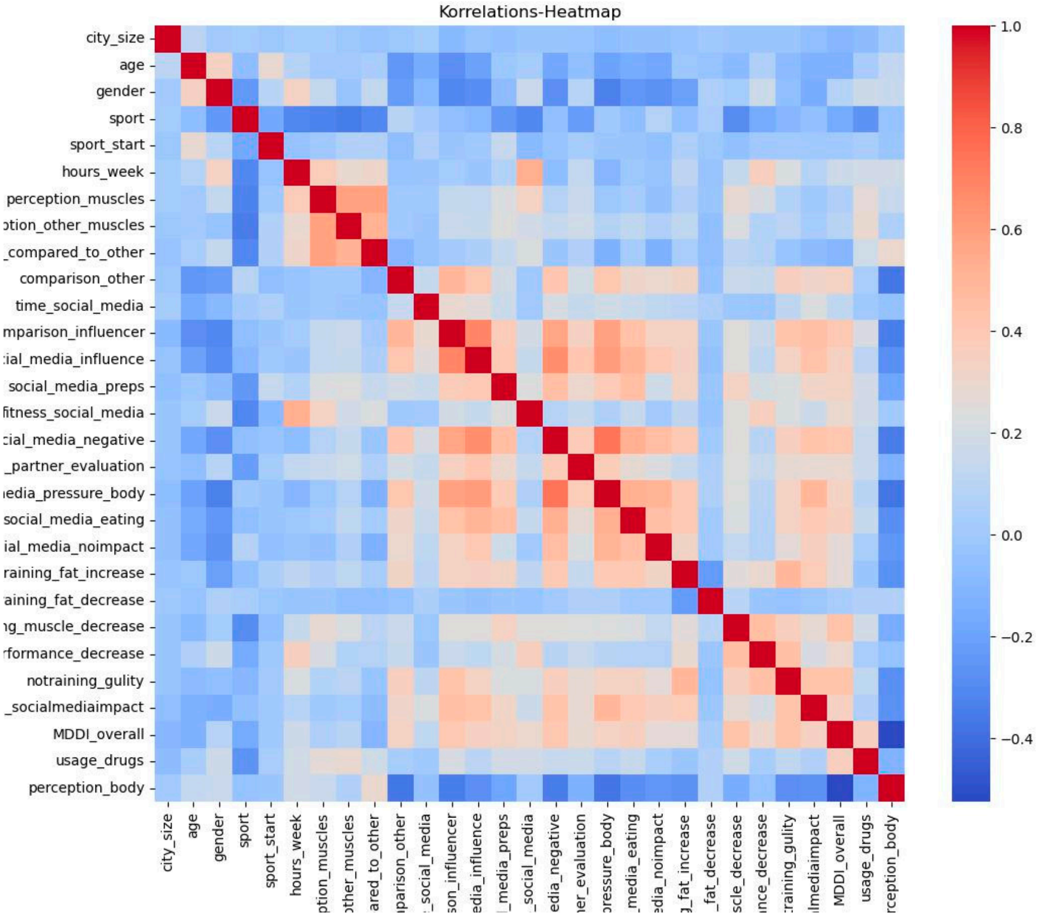

Supplementary Figure S2: Associations Between Social Media Use, Body Image, and Eating Behavior: Results of the Correlation Analysis The correlation heatmap revealed significant linkages among variables, notably those relating to social media's influence on body image, eating behavior, and comparisons with fitness influencers, showcasing strong correlations.
